# Differences between Patients with Sporadic and Familial Pheochromocytoma—Is It Possible to Avoid Genetic Testing in Certain Patients?

**DOI:** 10.3390/biomedicines12061352

**Published:** 2024-06-18

**Authors:** María Consuelo Muñoz, Beatriz Febrero, Miriam Abellán, Antonio Miguel Hernández, José Manuel Rodríguez

**Affiliations:** 1 Service of Endocrinology and Nutrition, Hospital Comarcal del Noroeste, 30400 Murcia, Spain; mconsuelo.munoz@gmail.com; 2 Endocrine Surgery Unit, General and Digestive Surgery Service, Hospital Universitario Virgen de la Arrixaca, 30120 Murcia, Spain; miriamabellanlu@gmail.com (M.A.); jmrodri@um.es (J.M.R.); 3 Department of Surgery, University of Murcia, 30120 Murcia, Spain; 4 Instituto Murciano de Investigación Biosanitaria Pascual Parrilla (IMIB_Pascual Parrilla), 30120 Murcia, Spain; 5 Service of Endocrinology and Nutrition, Hospital Universitario Virgen de la Arrixaca, 30120 Murcia, Spain; amiguel.hernandez@gmail.com; 6 Department of Medicine, University of Murcia, 30120 Murcia, Spain

**Keywords:** pheochromocytoma, genetic, sporadic, familial, index

## Abstract

Background: Pheochromocytoma (PHEO) is a rare neuroendocrine tumour with a strong genetic link, which therefore may modify its clinical behaviour and prognosis. The aim of the study is to evaluate the epidemiological and clinical differences between patients with sporadic and familial PHEO, as well as the specific differences in the index cases. Methods: A retrospective analysis of 136 patients in a tertiary hospital (1984–2021). Epidemiological, clinical, and histological variables were analysed. Statistics: SPSS 28.0 software was used. Univariate and multivariate logistic regression analyses were performed. *p* < 0.05 was considered statistically significant. Results: 64.71% of the cases (*n* = 88) presented a genetic mutation (familial cases). Additionally, 32.39% (*n* = 23) corresponded to index cases and the rest to screening cases. The main differences between patients with familial and sporadic PHEO were age (OR = 0.93 (0.89–0.97)), blood pressure-related symptoms (OR = 0.22 (0.06–0.89)), bilaterality (OR = 15.49 (3.76–63.84)), and size (OR = 0.70 (0.54–0.92)). Among patients with sporadic PHEO and index cases, only bilaterality was significant (OR = 13.53 (1.24–144.34)). Conclusions: Patients with familial PHEO diagnosed by screening differ from sporadic cases in terms of age, clinical features, and size. However, patients with sporadic PHEO only differ from index cases by a lower presence of bilaterality, which reaffirms the importance of genetic screening of patients with PHEO and their relatives.

## 1. Introduction

Pheochromocytoma (PHEO) is a rare neuroendocrine tumour. It differs from paraganglioma (PGL) by the site of origin; in the case of PHEO, the chromaffin cells of the adrenal gland are located in the adrenal medulla [1]. In the latest classification of the World Health Organisation (WHO) published in 2022, it is referred to as intra-adrenal PGL, as both are histologically the same. Histological examination cannot rule out malignancy, so all of them are considered potentially metastatic, and consequently, 10–25% are estimated to be metastatic [2,3].

Sporadic PHEO is diagnosed around the 4th–5th decade of life; however, familial cases with genetic mutations are diagnosed at an earlier age. Between 30 and 40% of cases have a genetic origin due to germline mutations. PHEO is the solid tumour with the greatest genetic predisposition, being the most frequent mutation in *Von Hippel Lindau* (*VHL*). For this reason, it is recommended that all patients undergo genetic screening [4,5,6,7,8,9,10].

Symptomatology is very varied, ranging from overt catecholaminergic symptoms to non-specific symptoms, depending on the biochemical phenotype and the continuous or paroxysmal release of catecholamines. It may also present with compressive symptoms, depending on the size [10]. In recent years, more and more asymptomatic cases are being diagnosed through incidentalomas or screening in patients carrying mutations, which means a variation in its classic form of presentation [10]. Despite this, the differences in the characteristics between familial and sporadic PHEO that may indicate the presence of a mutation before obtaining a genetic test result have not been analysed in detail. There is little literature establishing epidemiological, clinical, and histological differences between sporadic and familial PHEO to guide us in terms of clinical practice. If PHEO is caused by a mutation, it can change the management, prognosis, and follow-up of the patient, as well as the family counselling [8].

The objectives of the study are (1) to analyse the differences between patients with sporadic and familial PHEO; and (2) to analyse the differences between patients with sporadic PHEO and familial index cases.

## 2. Materials and Methods

### 2.1. Scope, Period, and Target Population under Study

A retrospective study of patients operated on and/or diagnosed with PHEO in a tertiary university hospital between 1984 and 2021.

For the diagnosis of FEO, the biochemical, radiological, and/or anatomopathological diagnosis was made as follows:

- Biochemical and radiological diagnosis: The biochemical diagnosis was carried out by 24 h urinary determination of catecholamines (adrenaline, noradrenaline, and dopamine), metanephrines, normetanephrines, and vanillyl mandelic acid. To confirm the biochemical diagnosis, an imaging test was performed, usually computed tomography (CT) or magnetic resonance imaging (MRI), and where necessary, nuclear imaging techniques such as ^123^-metaiodobenzylguanidine (^123^-MIBG) or ^68^-Gallium-DOTATOC (^68^Ga-DOTATOC) have been used. In the case of adrenal incidentaloma, the biochemical diagnosis was subsequent to the imaging diagnosis.
- Histopathologic diagnosis: diagnosis confirmed by the pathologist.

### 2.2. Patient Selection

The inclusion criteria were histological confirmation of PHEO and/or biochemical and radiological diagnosis, having a genetic test, a complete clinical history, and patients diagnosed and/or treated at the tertiary hospital.

### 2.3. Data Collection

Data were collected based on a retrospective review of the medical records of the FEOs treated at the HCUVA within the established period. A review of the evolution of these patients after diagnosis and treatment was carried out. 

Then, the patients who had to undergo the genetic study by Next-generation sequencing were summoned for information, signed informed consent, and a blood draw.

### 2.4. Genetic Analysis

All patients diagnosed with FEO who met the selection criteria were considered for genetic screening.

Next-generation sequencing (NGS) began to be marketed in 2005; it began to be used in our centre in 2017; therefore, genetic studies prior to this year were carried out by directed gene sequencing. Patients with known mutations who had developed PGL or PHEO at earlier ages in each family were also screened for NGS. A total of 60 patients were studied for NGS.

The genetic study was performed by NGS of all coding and splicing regions of a total of 11 genes involved in the development of tumours described in hereditary PGL/PHEO syndromes: *SDHA*, *SDHAF2*, *SDHB*, *SDHC*, *SDHD*, *MAX*, *TMEM127*, *VHL*, *NF1*, *RET*, and *FH*. In other patients, a gene genetic study was carried out according to the characteristics of the patient, and in cases with PGL or very early development of PHEO, the genetic study was repeated by NGS. In order to perform the genetic study, patients were first contacted and informed of the availability of genetic analysis with NGS; if they agreed, we obtained a signed consent form before obtaining the blood sample. All patient samples were analysed at the Clinical Biochemistry and Genetics Centre (CBGC) of the Hospital Clínico Universitario Virgen de la Arrixaca (HCUVA), as it is the reference centre for genetic analysis in the Region of Murcia, Spain.

The test was carried out by capture enrichment with specific probes (SureSelect QXT^®^ Agilent, Santa Clara, CA, USA) and subsequent sequencing on an Illumina device (Miseq, San Diego, CA, USA).

Following the genetic study, two groups were established: sporadic PHEO (without germline mutation) and familial PHEO. Familial PHEO is any pheochromocytoma that has a positive mutation, either as an index case (or de novo mutation) or those that were diagnosed by genetic screening. The index (or de novo) case is a patient diagnosed with a mutation without having a personal or family history of any of the mutations [11]. When a mutation is detected (index case or de novo mutation), all first-degree relatives are screened.

The study has been approved by the Ethics Committee of the HCUVA in 2023 under Code 2022-2-10-HCUVA.

### 2.5. Variables

The variables analysed to establish the differences between sporadic and familial PHEO were epidemiological (age and sex); side and bilaterality; symptoms: blood pressure (BP), cardiological (neurological and cutaneous); catecholaminergic profile (adrenergic, noradrenergic, dopaminergic, mixed, when a positive result could not be classified as pure, mixed, or normal). Among the mixed profiles, the mixed dopaminergic was defined as a tumour that produces dopamine combined or not with other catecholamines or their metabolites [12]. Histological variables included the PASS classification (Pheochromocytoma of the Adrenal gland Scaled Score), tumour size, and malignancy. 

### 2.6. Statistical Analysis

As far as the descriptive analysis of the sample is concerned, we used the number of present cases in each category and the corresponding percentage in the qualitative analysis, and for the quantitative analysis, the minimum, maximum, mean, and standard deviation values. 

Logistic regression modelling (univariate and multivariate) was used to determine the effect of epidemiological variables, clinical presentation, biochemical, and histological data on the association with familial and sporadic PHEO and index and sporadic PHEO patients. Multivariate models were evaluated using the Hosmer and Lemeshow test and the league table. Statistical analysis was performed using SPSS 28.0 for Windows. The differences considered statistically significant are those with a *p* < 0.05.

## 3. Results

### 3.1. Overall Description of the Series

**Figure 1:**
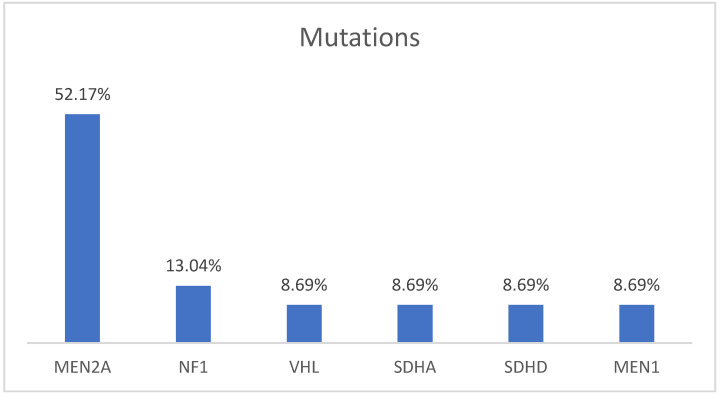
Distribution of germline mutations in index patients.

The initial sample included 192 patients diagnosed with PHEO, and the genetic study was performed on 136 patients (70.83%). Of the 136 patients included in the study, 64.71% (*n* = 88) presented a mutation: Multiple Endocrine Neoplasia type 2 (MEN 2A) in 86.36% (*n* = 76) with two different mutations of the *RET proto-oncogene*: Cys634Tyr in 75% (*n* = 66) and Cys634Arg in 11.36% (*n* = 10), Neurofibromatosis type 1 (NF1) in 3.41% (*n* = 3), Multiple PHEO syndrome and PGL in the SDHD subunit in 3.41% (*n* = 3), in the SDHA subunit, Multiple Endocrine Neoplasia type 1 (MEN 1), and VHL in 1.47% (*n* = 2), respectively. The index cases corresponded to 32.39% of the cases (*n* = 23), with which the following mutations were present: 52.17% (*n* = 12) MEN 2A, 13.04% (*n* = 3) NF1, 13.04% (*n* = 3) SDHD, 8.70% (*n* = 2) SDHA, 8.70% (*n* = 2) VHL, and 8.70% (*n* = 2) MEN 1 (Figure 1).

The mean age at diagnosis was 41.29 ± 14.84 years, and seventy-two patients (52.94%) were women. The number of left PHEOs was slightly higher than right PHEOs overall (52% versus 48%). The total number of bilateral PHEOs was 48.53% (*n* = 66). 

Regarding the clinic, 38.97% were asymptomatic (*n* = 53). Within the symptomatic group (*n* = 83), the most frequent symptoms were blood pressure-related in 37.5% (*n* = 51), cardiological in 25% (*n* = 34), neurological in 22.79% (*n* = 31), and cutaneous in 20.59% (*n* = 28) of cases. In addition, 52.51% (*n* = 71) of patients reported more than one symptom at diagnosis.

Concerning the catecholaminergic profile, 53 (38.97%) presented a pure profile, including adrenergic, noradrenergic, and dopaminergic secretion; 7 (5.15%) had normal catecholamine values; 14 (10.29%) had a positive value but were not classifiable; 2 (1.47%) had no catecholamines determined; and 57 (41.91%) had a mixed profile, with dopamine elevation in 40.35% of cases.

The mean size on histology was 3.80 ± 1.07 cm. Regarding the PASS classification, we obtained two different values, one for the first intervention and another for the group that underwent an intervention of the contralateral adrenal gland. The PASS score for the first intervention was 3.43 ± 2.35, and for the second intervention, it was 2.55 ± 1.67. Only two patients (1.47%) had a diagnosis of malignancy.

### 3.2. Differences between Sporadic and Familial PHEO

There is a statistically significant relationship between being male and having a familial PHEO (75% in cases of familial PHEO versus 25% for sporadic cases; OR = 2.40, *p* = 0.019) (Table 1). The mean age in sporadic cases was 50.39 ± 14.74 years and in familial cases 36.32 ± 12.39 years (Table 1), with a statistically significant difference (OR = 0.93, *p* < 0.001). 

Bilaterality also has a statistically significant link with familial PHEO (OR = 15, *p* < 0.001) (Table 1). 

Clinically, the results showed that the presence of BP-related symptoms, cardiological, neurological, and skin symptoms decreases the likelihood of having familial PHEO (OR = 0.15, *p* < 0.001; OR = 0.28, *p* = 0.001; OR = 0.38, *p* = 0.012; and OR = 0.34, *p* = 0.009, respectively) (Table 1). 

In the laboratory study, a lower probability of familial PHEO was observed in cases with a mixed secretion profile (OR = 0.41, *p* = 0.018) (Table 1).

With regard to the histological findings, the PASS scale did not show statistical significance, although there is a trend towards a higher score in cases of sporadic PHEO (3.95 ± 2.59 versus 3.04 ± 2.17) (Table 1). However, the size of the tumour presented a statistically significant relationship, correlating inversely in familial cases, with 4.55 ± 3.24 cm versus 3.04 ± 1.89 cm in diameter for sporadic and familial PHEO, respectively (OR = 0.79; *p* = 0.002) (Table 1). 

On the adjusted multivariate analysis, the variables that remained significant were age (OR = 0.93, *p* = 0.001), bilaterality (OR = 14.38; *p* < 0.001), BP-related symptoms (OR = 4.51; *p* = 0.034), and size (OR = 6.40; *p* = 0.011). Thus, if age or size increases or the patient presents BP-related symptoms, the likelihood of familial PHEO decreases. Conversely, bilaterality increases the probability of presenting a familial PHEO (Table 2).

### 3.3. Differences between Patients with Sporadic PHEO and Index Cases

When comparing the 48 sporadic PHEO cases with the 23 index cases, there were no statistically significant differences related to the patients’ gender (*p* = 0.242). Age showed a statistical association, with a decreasing probability of presenting a PHEO with a genetic mutation (index case) at an older age (OR = 0.94, *p* = 0.003). The age difference between both groups was 50.39 ± 14.7 years versus 37.61 ± 14.8 years for sporadic and index cases, respectively (Table 3). 

Bilaterality was more frequent in the index case group, being statistically significant (64.7% versus 35.3%; OR = 6.4, *p* = 0.002) (Table 3). 

Clinically, no statistically significant difference was demonstrated in the symptomatology associated with sporadic PHEO compared to index cases (Table 3). 

Regarding laboratory results, a statistically significant association was found between the mixed dopaminergic profile and index cases (OR = 7.56, *p* = 0.023) (Table 3). Eight patients out of the index cases had the following secretion profile: five patients with MEN 2A, one with VHL, one with SDHD without concomitant PGL, and one with NF1.

On histological findings, no statistically significant differences were found in the PASS score. As well as for a comparison between familial and sporadic PHEO, the PASS scale did not reach statistical significance; a higher score was observed in sporadic PHEO in this group, more striking with the index cases than in familial PHEO, 3.95 ± 2.59 versus 2.25 ± 1.50 (OR = 0.69; *p* = 0.222). In terms of size, both were very similar in sporadic and index cases, 4.55 ± 3.24 versus 3.85 ± 2.45 cm (Table 3). 

On the multivariate analysis, bilaterality was the only statistically significant variable (OR 13.35; *p* = 0.033) (Table 4).

## 4. Discussion

In recent years, there has been a rise in published articles on PHEO related to genetics, but very few of them compare the characteristics of sporadic and familial PHEO and, even more, differentiate between sporadic and index cases. 

With the genetic test, we have diagnosed a positive de novo mutation, as an index case, in 32.39% of patients. This percentage will increase as the NGS panel of each centre includes more genes and new mutations are discovered. The percentage of mutations that we have obtained is considerably higher than that published in other studies, such as Sbardella et al., which reached 11.7% [13]. It is even higher than described in studies that include PHEO and PGL, like Kim et al., where the mutation rate was 13.2% [14], or the studies of Guilmette and Shadow et al. [15], and Martins and Bulgalho et al. with 24% of the mutations [16]. Our rate is very similar to another Korean paper, which also included PGL, with a percentage of 32.6% [17]. 

The overall percentage of familial PHEO in our study rises to 64.71%, one of the highest prevalence rates in the literature. In a recent study published by Araujo-Castro et al. in which only PHEO was taken into account and PGL was excluded, the percentage of familial PHEO was 31%, even using a broader panel than ours that included 25 genes [4]. The predominant gene in our series was the proto-oncogene *RET* for MEN 2A, representing 86.36% of the 88 patients with a positive mutation, as there is a high prevalence of this syndrome in our geographical area. This high prevalence of the *RET* mutation has also been seen in other studies [4,18,19], in contrast to other published data that shows the VHL mutation as the most frequent, as well as the SDHB mutation [8,9]. As mentioned before, the most frequent mutation was *RET*, followed by *NF1* and *SDHD*, with a *VHL* mutation in two patients, similar to other series [4,13], and we do not have any patients with a mutation in SDHB. It is worth highlighting the two mutations in *SDHA*, as it is very rare and exceptional to develop a PHEO. The study, which was published by Ma et al., included seven patients with a mutation in *SDHA*, but only one developed a PHEO, and the rest were PGL. In the study of Araujo-Castro et al., there is also a case of PHEO with a mutation in *SDHA* [4,20].

With respect to gender, it is more frequent in women in both groups, the index cases and the familial group, but without a statistically significant difference, as has been described previously in other studies [21,22,23,24]. A particular finding is the association between being male and having a familial PHEO, with a probability 2.4 times higher, a fact not previously described in the literature. 

Sporadic PHEO is diagnosed between the 4th and 5th decade of life; in our study, the mean age in this group was 50.19 ± 14.7 years, similar to that reported before [8,10]. Previous studies showed that mutation-positive cases are younger than sporadic cases; in our study, the mean age of the index cases was 37.61 ± 14.8 years, without a significant statistical association in the multivariate analysis, which could be justified by the strong relationship between bilaterality and family character, with this variable appearing as significant in the univariate analysis. The study of Araujo-Castro et al. also stated that the younger the age, the higher the probability of having a mutation-positive PHEO, establishing age as a risk factor for a familial PHEO [4]. In the familial group, including screening cases, the mean age was 36.32 ± 12.39 years, similar to the index cases but with a lower standard deviation, obtaining statistically significant results in the multivariate analysis. This fact can be explained because, in these patients, an active search of the tumour is carried out even before the clinical manifestations appear, resulting in a diagnosis at an earlier age [8,9,19]. 

In addition to age, another criterion that should not guide us in carrying out a genetic study is the absence of family history, as there is a non-negligible percentage that may have a germline mutation despite there being no previous cases in the family. In the study published by Sbardella et al., 15.1% of patients with no family history had a positive mutation [13]. This is due to the low penetrance of some mutations, the type of inheritance, and the presence of de novo mutations [22,23]. 

Regarding the location of the tumour, there is a statistically significant association between having a bilateral PHEO and a positive germline mutation, both in index cases and in relatives. Having a bilateral PHEO increases the probability of having a positive genetic mutation, as previously described in the literature [4,14,17,25]. Therefore, it is noteworthy that six cases with bilateral PHEO have no mutation identified in our panel, hence the importance of expanding the genetic panel in these cases where there is a high suspicion of mutation.

In our series, we have a finding that differs from the rest of the publications: the tumour is more frequent on the left adrenal gland, specifically in sporadic cases of PHEO [25,26,27].

With regard to the clinical manifestations, 38.97% were asymptomatic (*n* = 53). Within the symptomatic group, the most frequent symptoms were BP-related in 37.5% (*n* = 51). Similar results were found in the study of PHEO in MEN 2A published by Rodríguez et al., with 51.8% of patients asymptomatic and hypertension the most frequent clinical characteristic in 50% of symptomatic patients, followed by palpitations and perspiration in 37.5% and 31.2%, respectively [24]. In relation to the differences between sporadic and familial cases, the index cases do not present statistically significant differences with any symptom compared with sporadic cases. However, if we also analyse the family screening cases, we do observe a greater presentation of different symptoms in the sporadic cases, especially those related to BP. The study by Araujo-Castro et al. states that patients with normotension are more likely to be associated with a mutation-positive PHEO [4], which supports our result.

In relation to the secretion profile, we found a lower probability of finding a familial PHEO when there is a mixed secretion, a result that has not been described in previous articles. It has been published that in familial cases there is a greater association in cluster 1, where we found SDH and VHL, with a noradrenergic profile, and in cluster 2, where we found MEN 2A and NF1, with an adrenergic profile [28]. In our familial patients, we have not been able to see the statistical association of these secretion profiles with familial cases, but a greater trend is observed. 

On the other hand, when analysing specifically the index cases, there was an association with the mixed dopaminergic profile in the univariate analysis. In a previous study published by our group, cases of dopamine-secreting PHEO in patients with *RET* protooncogene mutations were described without it being clear whether there was an association [25]. The dopaminergic profile has only been published in relation to sporadic PHEO and PGLs. In fact, Eisenhofer et al. concluded in their study that dopamine secretion was not characteristic of either VHL or MEN2A [27]. Dopamine production has also been associated with malignancy [1], but none of our patients with dopamine production have had a metastatic PHEO. Foo et al. reported that many diagnoses of dopaminergic secretion are missed because dopamine testing is not included in screening worldwide and concluded that it is advisable to request urinary or plasma dopamine in all patients with suspected PHEO or PGL [29]. In our case, this could be the explanation for having more dopamine-producing cases, as dopamine is included along with the rest of the catecholamines. 

As histological variables, we assessed the PASS scale and tumour size. The PASS scale had an important limitation, as it was calculated in few cases. On the one hand, it was developed in 2002, and tumours diagnosed previously did not have it calculated; on the other hand, in our histology department, it was not implemented unanimously. A trend towards a higher score was observed in sporadic cases. Other published studies, including Agarwal et al., concluded that the PASS scale cannot be reliably applied to PHEO for predicting malignancy [30], and Stenman et al. analysed the application of the PASS and GAPP scales in MEN 2A patients. They concluded that both scoring systems may be suboptimal for determining true malignant potential in PHEO with *RET* mutations and advocated for the restrictive use of these scores in MEN 2A cases until the results are replicated in larger numbers [31].

In tumour size, there are only differences in cases of familial PHEO, which shows a statistically significant relationship, while index cases do not. This can be explained by the fact that, as it occurred with age and clinic, using screening involves an active search for the tumour being thus diagnosed with a smaller size; these data can also be observed in another study [4]. Index cases are diagnosed in the same way as sporadic cases, so, in principle, the size should not be different. The fact of diagnosing a PHEO with smaller sizes gives us certain advantages in terms of patient management, as they should be less secretory, less symptomatic, have fewer postoperative complications, benefit from a laparoscopic approach, and present fewer cardiovascular complications [30].

The differences are more evident when comparing sporadic PHEO with familial cases because we include patients that are diagnosed by screening. When we compare sporadic PHEO and index PHEO, there is a trend towards a younger age and bilaterality. 

In this study, we had certain limitations. One of them is the scarcity of the literature on isolated PHEO without including PGL in order to have studies to compare our data, since due to its low prevalence, joint PHEO and PGL studies are usually carried out and the frequency and type of mutations vary from one tumour to another. In addition, as it was a retrospective study, some patients could not undergo the genetic study for different reasons; complete secretion profiles and PASS determination were not available. As a strength, it is an outstanding sample for a single-centre study in a region with a high prevalence of MEN 2A syndrome, which has allowed us to carry out a comparative analysis with an important sample of familial cases. 

## 5. Conclusions

The significant variables related to familial PHEO in our study are younger age, lack of symptoms, especially those related to blood pressure, and smaller size. However, if we compare index cases with sporadic cases, we only find differences in rates of bilaterality, with a positive trend towards younger ages. These results suggest that in the case of a patient with PHEO with no family history or lack of comorbidities characteristic of a hereditary syndrome, a genetic study should be carried out, as only bilaterality would point to a genetic origin.

Due to the low incidence of PHEO, these patients should be referred to specialised centres as they require specific management, including the necessity of genetic studies. Even in tertiary centres and geographical areas with high rates of PHEO, there are still many unknown mutations. As it is currently impossible to avoid the genetic study, it would be beneficial to increase the number of genes included in each panel to diagnose most familial PHEOs and better understand their specific characteristics in comparison to sporadic cases.

## Figures and Tables

**Table 1:** Differences between patients with sporadic and familial PHEO. Univariate analysis.

Variable	Sporadic	Family	OR (95% CI)	*p*-Value
Female sex	32 (44.4%)	40 (55.6%)	1	**0.019**
Male sex	16 (25%)	48 (75%)	2.40 (1.15–4.99)	
Age	50.39 ± 14.74 years	36.32 ± 12.39 years	0.93 (0.90–0.96)	**<0.001**
Bilaterality				**<0.001**
No	42 (60%)	28 (40%)	1	
Yes	6 (9.1%)	60 (90.9%)	15.00 (5.71–39.41)	
BP-related symptoms				**<0.001**
No	12 (16.4%)	61 (83.6%)	1	
Yes	36 (57.1%)	27 (42.9%)	0.15 (0.07–0.33)	
Cardiological symptoms				**0.001**
No	24 (25.8%)	69 (74.2%)	1	
Yes	24 (55.8%)	19 (44.2%)	0.28 (0.13–0.59)	
Neurological symptoms				**0.012**
No	27 (28.4%)	68 (71.6%)	1	
Yes	21 (51.2%)	20 (48.8%)	0.38 (0.18–0.81)	
Skin symptoms				**0.009**
No	30 (29.1%)	73 (70.9%)	1	
Yes	18 (54.5%)	15 (45.5%)	0.34 (0.15–0.77)	
Adrenergic profile				0.568
No	36 (35.6%)	65 (64.4%)	1	
Yes	9 (30%)	21(70%)	1.29 (0.54–3.12)	
Noradrenergic profile				0.915
No	38 (34.5%)	72 (65.5%)	1	
Yes	7 (33.3%)	14 (66.7%)	1.06 (0.39–2.84)	
Dopaminergic profile				0.644
No	44 (34.1%)	85 (65.9%)	1	
Yes	1 (100%)	1 (50%)	0.52 (0.03–8.48)	
Mixed profile				**0.018**
No	19 (25.7%)	55 (74.3%)	1	
Yes	26 (45.6%)	31 (54.4%)	0.41 (0.20–0.86)	
Normal profile				0.276
No	44 (35.5%)	80 (64.5%)	1	
Yes	1 (14.3%)	6 (85.7%)	3.30 (0.39–28.29)	
Mixed dopaminergic profile				0.42
No	17 (50%)	17 (50%)	1	
Yes	9 (39.1%)	14 (60.9%)	1.56 (0.53–4.55)	
PASS scale	3.95 ± 2.59	3.04 ± 2.17	0.85 (0.65–1.10)	0.213
Size	4.55 ± 3.24 cm	3.04 ± 1.89 cm	0.79 (0.67–0.92)	**0.002**

**Table 2:** Differences between sporadic and familial PHEO. Multivariate analysis.

Variable	OR	95% CI	*p*
Sex	2.17	0.65–7.26	0.208
Age	0.93	0.89–0.97	**0.001**
Bilaterality	15.49	3.76–63.84	**<0.001**
Blood pressure-related symptoms	0.22	0.06–0.89	**0.034**
Cardiological symptoms	0.45	0.12–1.73	0.243
Neurological symptoms	0.81	0.21–3.22	0.768
Skin symptoms	0.59	0.16–2.23	0.439
Mixed profile	0.67	0.20–2.19	0.502
Size	0.70	0.54–0.92	**0.011**

**Table 3:** Differences between patients with PHEO index cases and sporadic cases. Univariate analysis.

Variable	Sporadic	Index Cases	OR (95% CI)	*p*-Value
Female sex	32 (72.7%)	12 (27.3%)	1	0.242
Male sex	16 (59.3%)	11 (40.7%)	1.83 (0.67–5.06)	
Age	50.39 ± 14.7 years	37.61 ± 14.8 years	0.94 (0.90–0.98)	**0.003**
Bilaterality				**0.002**
No	42 (77.8%)	12 (22.2%)	1	
Yes	6 (35.3%)	11 (64.7%)	6.42 (1.97–20.96)	
BP-related symptoms				0.393
No	12 (60%)	8 (40%)	1	
Yes	36 (70.6%)	15 (29.4%)	0.63 (0.21–1.84)	
Cardiological symptoms				0.607
No	24 (64.9%)	13 (35.1%)	1	
Yes	24 (70.6%)	10 (29.4%)	0.77 (0.28–2.09)	
Neurological symptoms				0.983
No	27 (67.5%)	13 (32.5%)	1	
Yes	21 (67.7%)	10 (32.3%)	0.99 (0.36–3.52)	
Skin symptoms				0.63
No	30 (69.8%)	13 (30.2%)	1	
Yes	18 (64.3%)	10 (35.7%)	1.28 (0.47–3.52)	
Adrenergic profile				0.298
No	36 (65.5%)	19 (34.5%)	1	
Yes	9 (81.8%)	2 (18.2%)	0.42 (0.08–2.15)	
Noradrenergic profile			2.17 (0.63–7.53)	0.222
No	38 (71.7%)	15 (28.3%)		
Yes	7 (53.8%)	6 (46.2%)		
Dopaminergic profile		21 (32.3%)		
No	44 (67.7%)			
Yes	1 (100%)			
Mixed profile				0.441
No	19 (63.3%)	11 (36.7%)	1	
Yes	26 (72.2%)	10 (27.8%)	0.66 (0.24–1.88)	
Profile not classifiable				0.222
No	44 (69.8%)	19 (30.2%)	1	
Yes	1 (33.3%)	2 (66.7%)	4.63 (0.40–54.21)	
Normal profile				0.584
No	44 (68.8%)	20 (31.3%)	1	
Yes	1 (50%)	1 (50%)	2.20 (0.13–36.97)	
Mixed dopaminergic profile				**0.023**
No	17 (89.5%)	2 (10.5%)	1	
Yes	9 (52.9%)	8 (47.1%)	7.56 (1.32–43.37)	
PASS scale	3.95 ± 2.59	2.25 ± 1.50	0.69 (0.38–1.26)	0.222
Size	4.55 ± 3.24 cm	3.85 ± 2.45 cm	0.92 (0.77–1.10)	0.368

**Table 4:** Differences between patients with PHEO index cases and sporadic cases. Multivariate analysis.

Variable	OR	95% CI	*p*-Value
Age at diagnosis	0.93	0.86–1.01	0.083
Bilateral	13.35	1.24–144.34	**0.033**
Mixed dopaminergic profile	1.33	0.12–14.50	0.817

## Data Availability

Data is contained within the article.
